# T128. THE ASSOCIATION BETWEEN GENETIC RISK FOR SCHIZOPHRENIA AND PATTERNS OF CIGARETTE AND CANNABIS USE IN ADOLESCENCE

**DOI:** 10.1093/schbul/sby016.404

**Published:** 2018-04-01

**Authors:** Stanley Zammit, Hannah Jones, Suzanne Gage, Jon Heron, George Davey Smith, Glyn Lewis, Michael O’Donovan, Michael Owen, James Walters, Marcus Munafò

**Affiliations:** 1 Cardiff University; 2 University of Bristol; 3 University of Liverpool; 4 University College London

## Abstract

**Background:**

Schizophrenia is associated with a higher prevalence of cannabis use and cigarette use. However, it is unknown to what extent these associations are due to a shared genetic aetiology. We therefore aim to examine how schizophrenia genetic risk associates with patterns of cigarette and cannabis use in adolescence.

**Methods:**

We analysed repeated measures of cigarette and cannabis use during adolescence in a sample of 5,300 individuals in the Avon Longitudinal Study of Parents and Children (ALSPAC) birth cohort who had at least 3 measures of cigarette and cannabis use between ages 14–19 years. Cigarette and cannabis use data were summarised using longitudinal latent class analysis to identify longitudinal classes of substance use, and associations between polygenic scores for schizophrenia and resulting classes were assessed.

**Results:**

The schizophrenia polygenic score based on single nucleotide polymorphisms (SNPs) meeting a discovery sample threshold of p ≤ 0.05 was associated with late onset cannabis use as compared to non-use (OR = 1.20; 95% CI = 1.05, 1.37) but not with early onset or cigarette only use latent classes. This association persisted after excluding the CHRNA5-CHRNA3-CHRNB4 nicotinic receptor gene cluster (OR = 1.25; 95% CI = 1.08, 1.44), a locus which has previously been found to strongly associate with schizophrenia.

**Discussion:**

This study found that genetic risk of schizophrenia (as captured by polygenic scores) is associated with late-onset cannabis use but not with other smoking phenotypes in adolescence in ALSPAC. Possible explanations for these results are that schizophrenia and cannabis use have a shared genetic aetiology or that biological risk of schizophrenia leads to cannabis use through secondary mechanisms. These secondary mechanisms may include stress of childhood behavioural problems occurring as a result of biological processes underling schizophrenia. Future analyses involving mediation models may shed some light on factors influencing patterns of substance use in individuals with a high genetic liability for schizophrenia.

